# Enhancing knee MRI bone marrow lesion detection with artificial intelligence: An external validation study

**DOI:** 10.1016/j.redii.2025.100063

**Published:** 2025-08-14

**Authors:** Kevin Maarek, Philippine Cordelle, Tom Vesoul, Pascal Zille, Gaspard d'Assignies, Antoine Feydy, Guillaume Herpe

**Affiliations:** a Université de Paris, 85, boulevard Saint-Germain, 75006 Paris, France; b Incepto Medical, Paris, France; c Groupe hospitalier du Havre, 76083 Le Havre, France; d Hôpital Cochin, 27, rue du Faubourg-Saint-Jacques, 75016 Paris, France; e CHU de Poitiers, 2, rue de la Milétrie, 86000 Poitiers, France

**Keywords:** Radiology, Bone marrow edema, Osteoarthritis, Artificial intelligence, Retrospective study

## Abstract

**Background:**

Magnetic resonance imaging (MRI) is a sensitive imaging modality for identifying knee bone marrow edema, a significant biomarker in osteoarthritis and injury assessment. The precision of bone marrow edema detection is contingent upon the radiologist's expertise, and segmentation efficiency demands substantial time.

**Purpose:**

This study evaluated artificial intelligence's (AI) impact on enhancing general radiologists' diagnostic accuracy for bone marrow edema detection in knee MRI.

**Materials and methods:**

A multicenter, multireader, multicase methodology was used in this retrospective diagnostic study, which relied on an external dataset of 198 examinations. Mean age was 46 years with a standard deviation (SD) of 15.8 years and a female/male ratio of 49 %/51 %.

An AI algorithm from the AI solution Keros, comprising three orientation-specific 3D-UNet models, was deployed for bone marrow edema segmentation on T2/proton density with fat suppression sequences.

The ground truth was set by expert musculoskeletal radiologists.

The purpose was to externally validate the AI algorithm and compare the performance and speed of bone marrow edema identification by less experienced radiologists when using the algorithm versus not using it

**Results:**

A total of 184 patients were included. With AI, readers’ sensitivity for bone marrow edema detection significantly increased by 6.1 % from 79.3 % without AI (95 % confidence interval [95 % CI]: 77.2–80.3 %) to 85.4 % (95 % CI: 84–86.2 %) with AI (*p* = 0). Specificity significantly increased by 5 % with AI assistance, reaching 93.9 % (95 % CI: 93.7–94.6 %) from 88.9 % (95 % CI: 88.6–89.4 %) (*p* = 0). Reading times were reduced by 42 % (0.66 min per exam, *p* = 3.81e-41).

**Conclusion:**

AI significantly increased the sensitivity and specificity of bone marrow edema detection for general radiologists and shortened the reading process. AI-assisted detection of bone edema in the knee also opens up new perspectives for the longitudinal monitoring of patients with knee osteoarthritis.

## 1 Introduction

Degenerative, or post-traumatic knee disorders have a major impact on patients' quality of life and represent a significant economic cost for society [1], [2], [3], [4]. Magnetic resonance imaging (MRI) is a sensitive imaging modality for both diagnosing and monitoring knee conditions, providing diagnostic and prognostic insights [5].

Bone marrow edema is an important feature on MRI in musculoskeletal disorders [6]. Bone marrow edema is often associated with various knee pathologies, it can either be a temporary and self-resolving condition in bone contusions or algodystrophy, or it may indicate significant structural damage with therapeutic implications in conditions like gonarthrosis, rheumatoid arthritis, or spontaneous osteonecrosis [7], [8], [9], [10], [11], [12]. In such cases, bone marrow edema is indicative of an advancement in inflammatory processes and pathological structural alterations.

Bone marrow edema is defined as increased signal intensity on T2-weighted (T2-W) images with fat suppression with corresponding low signal on T1-weighted (T1-W) sequences and is often diffuse and ill-defined. Given that MRI and dual energy computed tomography (CT) are the exclusive imaging techniques capable of identifying bone marrow edema, it is possible that cases of this disorder may go undetected when knee pain is investigated by X-ray, ultrasound or CT [13].

In addition, considering the importance of bone marrow edema in the clinical progression and monitoring of knee conditions there is a growing demand for both precise qualitative and quantitative evaluation [14], [15], [16], [17].

The diagnostic efficacy of knee MRI is contingent upon the subspecialty expertise of the interpreting practitioner and their cumulative years of imaging experience [18,19].

Nowadays, the identification and grading of bone marrow edema is carried out through manual examination, and evaluated qualitatively by a radiologist which leads to varying accuracy levels depending on reader expertise [20].

Because of the substantial workload and the constraints associated with existing segmentation tools, quantitative methods involving manual segmentation of bone marrow edema are employed in research settings but are not integrated into clinical practice.

Artificial intelligence (AI) algorithms have, over the last few years, shown increasing performances in difficult segmentation tasks especially in knee imaging [21].

This study aims to evaluate the impact of AI assistance on the diagnostic accuracy of radiologists for detecting bone marrow edema in knee MRI.

## 2 Material and methods

Data analysis and manuscript writing were partially performed by authors affiliated with Incepto Medical.

### 2.1 Study design

**Fig. 1:**
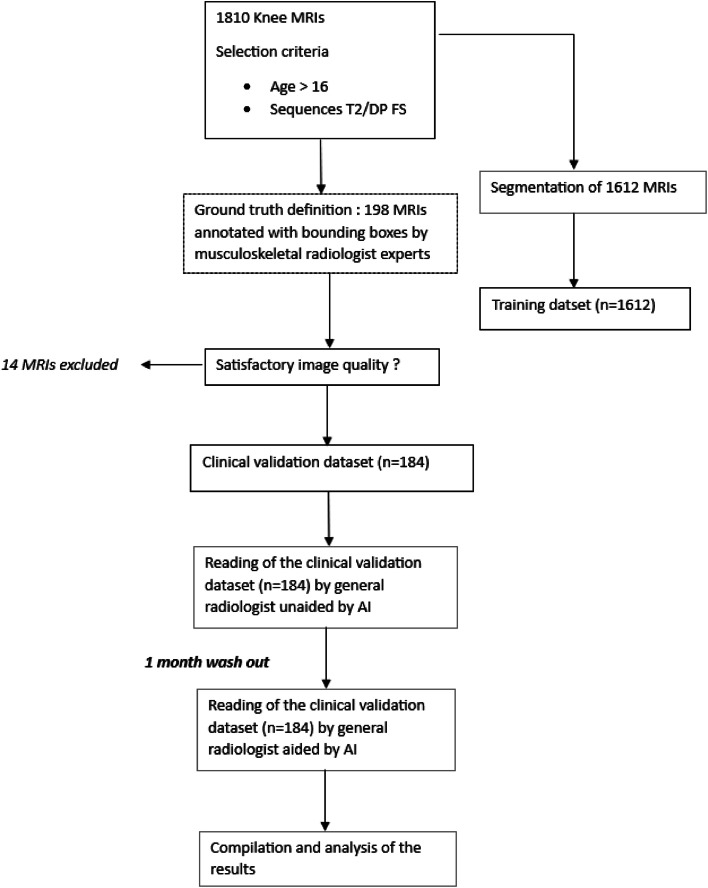
Retrospective study to evaluate the impact of artificial intelligence (AI) assistance on the diagnostic accuracy of radiologists for detecting bone marrow edema in knee MRI: Overall study deisgn. DP: proton density; FS: fat suppression; MRI: magnetic resonance imaging.

The current study is a retrospective diagnostic study using the multireader, multicase methodology [22], based on an external multicenter data set. The overall study design is summarized in Fig. 1.

### 2.2 Data collection

All clinical sites adhered to the identical study protocol. DICOM images were anonymized. To construct the training dataset, 1612 knee MRI studies were retrospectively gathered from three private imaging groups comprising 15 centers in France and Switzerland. These MRI scans were part of daily routine screenings and included a variety of clinical indications. Both post-traumatic and degenerative MRIs were used. The dataset comprised normal MRIs without any visible bone marrow edema, MRIs showing subtle bone marrow edema, and MRIs with severe and clearly marked bone marrow edema. The diversity in the training data allowed the model to generalize effectively and improved its ability to detect both early and advanced stages of bone marrow edema. The training and validation datasets were not subjected to a precise quantification of bone marrow edema.

A healthy volunteer cohort was not included.

### 2.3 AI model

#### 2.3.1 Training data-set description

The AI algorithm was trained on a dataset of 1612 patients (50.9 % female, 49.1 % male). Mean age was 46.2 with a standard deviation (SD) of 18.4 years. The inclusion criteria for this study stipulated that only individuals aged 16 years and above were eligible for processing using the AI software Keros v 2.0 (Incepto Medical, Paris, France).

Details regarding the population and MRI manufacturer used in the training dataset can be found in Tables S1 and S2 in Appendix A.

#### 2.3.2 Training procedure: segmentation

In order to train the algorithm to detect bone edemas, neural networks were actually trained to segment them. Data scientists created the training masks by using an in-house annotation tool, Genesis, with guidance from two senior radiologists (with 10 and 20 years of experience in musculoskeletal radiology), to segment by hand bone marrow edema within the training dataset. The input data consisted of axial, coronal, and sagittal T2- or proton density-weighted fat-saturated volumes (T2/PD fatsat).

The segmentation process involved three 3D-UNet models, each tailored to a specific orientation, known as UNet3D, and specifically trained for bone marrow edema segmentation. These models processed individual T2/PD-fatsat sequences, generating three-dimensional (3D) probability maps. Within these maps, each voxel's value indicated the likelihood of it belonging to a bone marrow edema.

#### 2.3.3 Training procedure: from a probability map to a binary segmentation

**Fig. 2:**
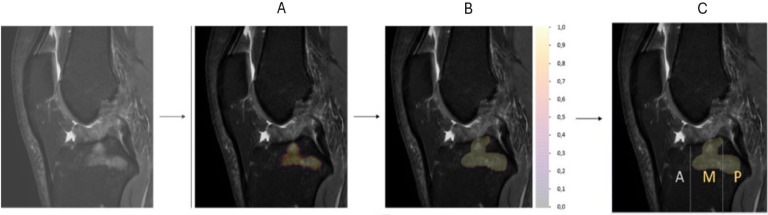
Retrospective study to evaluate the impact of artificial intelligence assistance on the diagnostic accuracy of radiologists for detecting bone marrow edema in knee MRI: Conversion of the probability map into isolated shapes (discrete probability map) and allocation to subregions. A: Initial probability map detecting bone oedema, where each pixel represents the likelihood of oedema presence. B: Segmentation of isolated shapes extracted from the probability map, converting the continuous map into discrete objects representing suspected oedema areas. C: Allocation of the segmented shapes to specific tibial subregions, here the middle and posterior medial tibia, allowing precise localization of bone oedema.

Following this, clear-cut bone edema segmentations were derived from the continuous probability map (depicted in Fig. 2). To outline edema contours, adapted thresholds specific numerical values tailored to the data characteristics — were applied, resulting in a stepped 3D probability map. In this map, non-edematous background voxels were null, and all voxels within the same edema shared identical probability values.

**Fig. 3:**
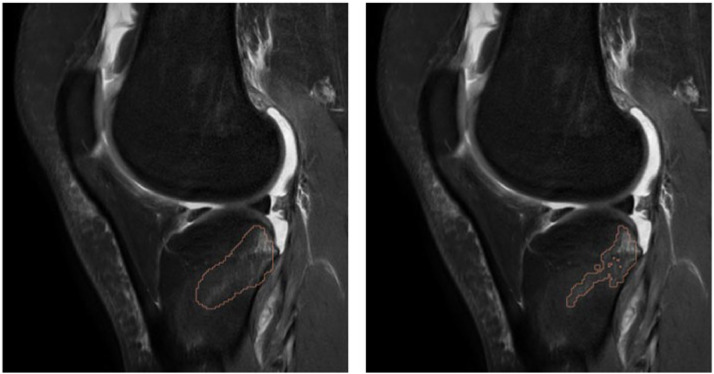
Retrospective study to evaluate the impact of artificial intelligence assistance on the diagnostic accuracy of radiologists for detecting bone marrow edema in knee MRI: Detection and segmentation of bone marrow edema by Keros software.

To improve the specificity of edema segmentation, post-processing algorithms refined the contours by retaining only the brightest voxels and discarding detections distant from cartilage, as shown in Fig. 3. This is based on evidence that clinically significant bone marrow edema in osteoarthritis typically occurs near the subchondral bone [23]., adjacent to cartilage, whereas hyperintensities in central medullary regions often reflect residual red marrow, especially in younger individuals. Accordingly, we implemented a distance-based confidence weighting strategy: the model’s output confidence for each edema voxel was linearly reduced with increasing distance from the nearest cartilage surface. This reduction followed a linear function, meaning that the farther the voxel was from the cartilage, the more its predicted confidence was down-weighted.

Comparison of bone marrow edema (BME) detection on knee MRI scans before and after post-processing. The left image shows the initial segmentation of BME areas, while the right one shows the refined segmentation generated by post-processing software. The refinement process retains only the most intense signal regions, corresponding to the highest probability of true edema, thereby improving specificity in the detection.

This helped suppress false positives due to central marrow signal abnormalities and focus detection on pericartilaginous, clinically relevant regions.

#### 2.3.4 Training procedure: region classification

The last stage of the pipeline aimed at transforming the segmentation result into a region classification. In previous studies exploring bone marrow edema quantification, the knee bones were divided in different articular subregions. In our study, the knee bones were divided into 15 subregions following several criteria from the MRI Osteoarthritis Knee Score (MOAKS) classification [23].

The semi-quantitative MOAKS is a widely used and well-validated instrument for evaluating knee osteoarthritis and has been applied in large-scale epidemiological studies such as the Osteoarthritis Initiative (OAI) [23,24].

**Fig. 4:**
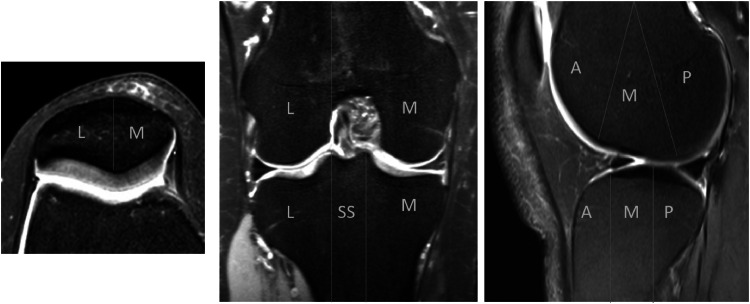
Retrospective study to evaluate the impact of AI assistance on the diagnostic accuracy of radiologists for detecting bone marrow edema in knee MRI: The 15 subregions of the knee bones, defined according to MOAKS classification criteria. In the leftmost image, the patella is divided into two subregions. In the middle and right images, both the femur and tibia are each divided into three subregions for the medial and lateral compartments.

The patella is divided into two subregions: the medial and lateral patella. The femur and tibia are divided into three medial (anterior, middle and posterior) and three lateral (anterior, middle and posterior) sub-regions. The subspinous subregion is delineated by the tibial spines. The 15 knee bones sub-regions are illustrated in Fig. 4.

Each edema is then allocated to one or multiple subregions to get a classification output.

For each edema, we calculate the proportion of its volume included in each subregion. The edema is then assigned to a sufficient number of subregions to cover at least 95 % of its volume. The final output provides a probability for each subregion: 0 if no edema is included, otherwise the maximum of the probabilities of the included edemas.

### 2.4 Validation data set acquisition

To construct the validation dataset, 198 knee MRI studies (distinct from the training data set) were retrospectively gathered from three private imaging groups comprising 15 centers in France and Switzerland.

These MRI scans were part of daily routine screenings, with a variety of clinical indications.

The inclusion criteria for this study included all consecutive individuals aged of 16 years and above.

The exclusion criterion was the presence of metallic artifact.

Examinations were issued from three different manufacturers Philips Healthcare (Amsterdam, Netherlands), GE Healthcare (Chicago, United States) and Siemens Healthcare (Erlangen, Germany) and three different magnetic fields (1, 1.5 or 3 T). All knee MRI examinations, acquired in clinical routine, had at least standard sequences: coronal, axial, and sagittal (either bidimensional [2D] or 3D with 2D reformatted images) fat suppressed proton density-weighted or fat-suppressed T2- weighted sequences.

MRI characteristics and acquisition parameters of the validation dataset are detailed in Table S2 of Appendix A.

The comorbidities of the patients of the validation test were recorded using electronic patient record.

### 2.5 Set-up for bone marrow edema detection

Annotators and experts used an in-house DICOM image annotator tool (Genesis, Incepto Medical, Paris, France) using multiplanar reformatting (MPR) which was very similar to the clinical picture archiving and communication system (PACS) environment usually used in clinical practice. Annotations were performed outside any clinical practice environment and clinicians had no time limit.

### 2.6 Ground truth definition

The ground truth was determined through the assessments of two board-certified, fellowship trained, musculoskeletal radiologists, each with over 10 years of experience.

They independently annotated without time limit 190 out of the 198 examinations using bounding boxes to highlight all instances of identified bone marrow edema. Eight examinations were not annotated by the experts due to poor quality (missing sequence, artifacts, loading issues).

They did not have access to clinical information and were blinded to the AI results.

The bounding boxes were then assigned to one or multiple subregions to achieve binary classification across the 15 subregions.

Examples of bone marrow edema annotated by both experts are given in Fig. S1 of Appendix A.

Experts recognized and labeled subchondral geodes as bone marrow edema. They excluded cystic lesions observed in the tibial spines from their annotations.

### 2.7 Radiologists for bone marrow edema detection

Three radiology residents, each with varying levels of experience (between two and five years), interpreted examinations to detect bone marrow edema in knee MRI scans.

The three readers were presented the validation data set twice: initially without AI software assistance and subsequently with AI assistance, with a one-month washout period between the two assessments.

The readers were initially trained in using the Genesis annotation platform through an instructional guide and then via a video conference training session. Following that, they underwent training in bone marrow edema annotation through annotating a separate set that included ten MRI exams distinct from the validation set.

Each annotators had a different shuffled worklist, and they did not communicate about the cases.

The annotators used the same annotation software as the experts. During the interpretation period with AI assistance, the bone marrow edema detected by the AI was marked on the DICOM images with bounding boxes. Along with the figure, prefilled report was provided to annotators.

Illustration of the Genesis annotation platform displaying three proton density-fat suppressed sequences and the form to fill out with the detected bone edemas is given in the Fig. S2 of Appendix A.

### 2.8 Reader performance

#### 2.8.1 Performances

Performances evaluation consisted in calculation of sensibility (percentage of correct predictions on pathological cases), specificity (percentage of correct predictions on healthy cases),​ positive predictive value (percentage of correctly identified positive cases), negative predictive value (percentage of (correctly identified negative cases), Accuracy (proportion of correctly identified cases (both true positives and true negatives) among the total number of cases) and area under the curve (AUC).

#### 2.8.2 Time measurement

To assess the reading time, we defined the time to report as the duration from the first opening of the study to the validation of the case. We measured it using a dedicated software within the annotation tool. Annotators were blinded to the time evaluation.

### 2.9 Statistical analysis

Statistical analysis was performed using the software Python.

Ninety-five percent confidence intervals were calculated for sensitivity, specificity, and AUC.

Since paired samples were employed in this statistical analysis (using the same exams for both phases), a McNemar statistical test was applied to compute p-values between the phase without AI and the phase with AI.

Intraclass correlation (ICC) values were calculated using the python pingouin library. We used the ICC (2, k) corresponding to two-way random effects, absolute agreement, multiple raters/measurements” ICC described by McGraw and Wong [25].

To assess time savings per examination between the AI-free session and the AI-assisted session, a Wilcoxon test was employed.

A significance level of *p* < 0.05 was deemed as statistically significant.

No imputation was made for missing data.

## 3 Results

### 3.1 Validation data set characteristics

One hundred and ninety-eight patients who had undergone knee MRI for chronic pain (50.4 %) and knee trauma (49.6 %) were eligible for the study. Table 1 presents the demographic details of the dataset alongside other abnormal findings in knee MRI.

**Table 1:** Results of a retrospective study to evaluate the impact of artificial intelligence assistance on the diagnostic accuracy of radiologists for detecting bone marrow edema in knee MRI: Validation data set characteristics.

Age and gender		Percentage (number)
Male		49 % (95)
Female		51 % (102)
Other		0.5 % (01)
Mean age		46 years (SD : 15.8 years)

Knee pathologies		Percentage (number)
Patellofemoral cartilage lesion		38.8 % (77)
Medial tibial-femoral cartilage lesion		30.8 % (61)
Lateral femorotibial cartilage lesion		13.6 % () (27)
Medial meniscus lesion		43 % (85)
Lateral meniscus lesion		17.7 % () (35)
Significant extrusion of medial meniscus		11.1 % () (22)
Significant extrusion of lateral meniscus		3.5 % () (7)
Anterior cruciate ligament injury		26.2 % (52)
Medial collateral ligament injury		13.1 % () (26)

Among them, MRI scans from 14 patients were excluded due to low image quality, such as metallic artifacts. Consequently, the analysis included a total of 184 patients.

A significant proportion of these patients presented with osteoarthritis lesions and meniscal injuries. A lesser percentage of these patients experienced ligament injuries.

Seven percent of MRI scans showed no abnormalities. 36 % of the MRIs were interpreted as pathological but did not exhibit bone marrow edema.

Across all bounding boxes annotated by the two experts, 93 % were marked as bone edema and 7 % as geodes. The intraclass correlation among the two experts was 0.88 (CI: 0.87, 0.89).

These abnormal findings were independently generated for this research study using the results from Keros 2.0, an AI-powered diagnostic tool developed by Incepto Medical. This software automatically analyzes knee MRI scans to detect and characterize a wide range of abnormalities, including bone marrow edema, ligament tears, meniscal lesions, cartilage defects, joint effusions, and popliteal cysts.

### 3.2 Main analysis

The metrics for all readers are compared in Table 2.

**Table 2:** Results of a retrospective study to evaluate the impact of artificial intelligence (AI) assistance on the diagnostic accuracy of radiologists for detecting bone marrow edema in knee MRI: Diagnostic performance of readers with and without AI assistance.

Parameter	Without AI ( %, [95 % CI])	With AI ( %, [95 % CI])	Delta ( %)
Sensitivity	79.3 [77.2, 80.3]	85.4 [84, 86.2]	+ 6.1 *p* = 0
Specificity	88.9 [88.6, 89.4]	93.9 [93.7, 94.6]	+ 5.0 *p* = 0

**Fig. 5:**
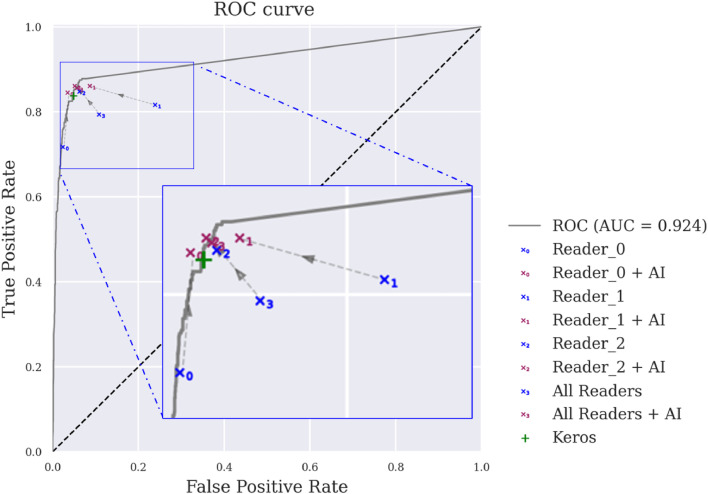
Results of a retrospective study to evaluate the impact of AI assistance on the diagnostic accuracy of radiologists for detecting bone marrow edema in knee MRI: Receiver operating characteristics (ROC) curve comparing generalist radiologist performances without and with AI aid and Stand Alone AI (Keros). AUC: area under the curve; AI: artificial intelligence.

The performance of generalist radiologists, both without and with AI assistance, along with Stand Alone AI, is depicted in Fig. 5.

### 3.3 Subgroup analysis

AI-aided reading showed a non-significant improvement or equal sensitivity for bone marrow edema detection across 13 out of 15 anatomic locations (with an observed improvement greater than 5 % in five subregions; *p* > 0.05, as detailed in Table S4 of Appendix A).

AI-aided reading showed improved or equal specificity for bone marrow edema detection across 14 out of 15 anatomic locations (with an observed improvement greater than 5 % in nine subregions; *p* < 0.05). Results are detailed in Table S4 of Appendix A.

### 3.4 Intraclass correlation

The intraclass correlation among the three readers significantly increased from 0.73 (95 % CI: 0.66, 0.77) without to 0.89 (95 % CI: 0.89, 0.90) with AI assistance (*p* < 0.001), thereby exceeding the ICC of 0.88 (95 % CI: 0.87, 0.89) observed between the two experts.

### 3.5 Reading time analysis

The reading time without AI was estimated at 1.59 min/exam (SD = 0.66 min) and 0.93 min/exam (SD =0.49 min) with AI aid. AI shortened the average reading time by 0.66 min (AI effect of −42 %) per examination (*p* < 0.001).

### 3.6 Evaluation of stand-alone AI performance

Regarding stand-alone AI performances, the area under the receiver operating characteristics (ROC) curve (AUC) was 0.92, the predictive positive value was 0.69, the negative predictive value was 0.97, the specificity was 0.95 and the sensitivity was 0.83.

Stand alone AI achieved AUC equal to or exceeding 0.92 across 10 out of 15 anatomic regions. Stand Alone AI performance results are detailed in Table S5 of Appendix A.

## 4 Discussion

MRI holds a crucial role in the diagnosis of knee pathologies, with bone marrow edema emerging as a significant indicator. While not pathognomonic for any specific disorder, its high sensitivity significantly influences diagnostic decisions, emphasizing the need for accurate detection.

Our results show that the high sensitivity and specificity of an automatic detection approach can improve radiologists’ performances, speed up the diagnostic process and reduce interobserver variability.

Given the growing incidence of degenerative knee pathologies resulting from demographic changes , particularly the aging of the Western population, and the high volume of knee MRIs performed, this study holds great significance [26].

Two former academic studies developed AI models for detecting bone marrow edema on MRI scans, with one achieving a 70 % detection sensitivity using a single 3D T2 sequence [21].

A recent article traced the 30-year evolution of semi-quantitative MRI in osteoarthritis research, outlining the potential impact of AI [27]. Published by the Osteoarthritis Research Society International in 2024, the study introduces various scoring systems, such as Whole-Organ Magnetic Resonance Imaging Score (WORMS) and MRI Osteoarthritis Knee Score (MOAKS), emphasizing SQ assessment's validity, reliability, and role in understanding osteoarthritis and treatment response. The Kellgren and Lawrence, a radiogrpahic grading system, developped in 1958, is still employed to diagnose structural osteoarthritis by assessing various features [28]. Despite advancements, radiographic joint space narrowing remains the primary outcome for demonstrating efficacy in phase III clinical trials [29]. However, recent work suggest that the inclusion of bone marrow edema, which can complement traditional imaging methods, may address limitations in fully capturing osteoarthritis progression compared to using radiography alone [30], [31].

Detection of bone marrow edema in patients with osteoarthritis could aid to identify individuals susceptible to symptomatic and structural disease progression [32]. Moreover bone marrow edema can potentially guide tailored therapeutic approaches [33]. The evolving understanding of the lesions' structural diversity implies varying responses to osteoarthritis-modifying drugs based on distinct phenotypes, such as meniscus-cartilage, subchondral bone, and inflammation, with the latter two phenotypes closely linked to bone marrow edema [34].

Additionally, bone marrow edema hold the potential to function as biomarkers for early detection of responses to interventions aimed at addressing related pathology [35], [36]. Therefore, automated evaluation of osteoarthritis bone marrow edema may enhance patient categorization by recognizing a subgroup of patients responsive to treatment, and then facilitate the assessment of treatment effectiveness [37].

Our study has several limitations. First, we did not apply predefined criteria for selecting the study population. However, the training and validation datasets were composed of knee MRI scans acquired during routine clinical practice across multiple institutions, which naturally ensured a diverse representation of knee pathologies, as reflected in Table 1.

Additionally, we did not perform a precise quantification of bone marrow edema, such as using a standardized scoring system like MOAKS. Incorporating such assessments could provide a more detailed evaluation of bone marrow edema distribution and severity in future work.

Although the T1 sequence was omitted from algorithm training, bone marrow edema detection, indicating increased water content, typically relies on sequences like T2-weighted, short tau inversion recovery (STIR), or proton density, which were used in training.

Our study employed a consensus ground truth based on MRI interpretation by two radiologists, without resolving discrepancies with a third party. Discrepancies may be attributed to false positives or negatives within the bounding box around a bone marrow edema. A wider bounding box could lead to false positives covering multiple subregions. To address disparities, all observations marked by either or both experts were included.

One limitation is selection bias, as the readers used the algorithm solely to identify bone marrow edema, which doesn't reflect a "real-world" scenario.

Additionally, we acknowledge the distinct histopathological nature of subchondral geodes compared to bone marrow edema. However, we chose to group them as bone marrow edema during the reading exercise because they share key imaging characteristics on MRI, such as increased signal intensity on fluid-sensitive sequences, which can challenge even expert readers in distinguishing between the two entities. Moreover, from a pathophysiological perspective, bone marrow edema and subchondral geodes can be considered as stages along a continuum of osteoarticular damage. Bone marrow edema often represents an early inflammatory response within the bone marrow, associated with hyperemia and increased vascular permeability. Over time, persistent inflammation and mechanical stress can lead to focal bone resorption and cystic changes, resulting in the formation of subchondral geodes. Therefore, including both entities under the bone marrow edema umbrella during the reading exercise reflects their overlapping imaging features and pathophysiological linkage, while also aligning with the clinical challenge of differentiating them on routine MRI.

## 5 Conclusion

MRI artificial intelligence assistance improves the sensitivity and may even improve the specificity of bone marrow edema detection by radiologists. It also reduces the time needed to interpret knee MRI. AI-assisted detection of bone edema in the knee also opens up new perspectives for the longitudinal monitoring of patients with knee osteoarthritis.

## Patient consent

Our multicentric institution has a general consent form signed by each patient to allow or refuse retrospective data analysis for research purposes. DICOM images were anonymized.

## Funding

This retrospective study received funding from Incepto Medical, the developer of the AI software Keros.

## Institutional review board approval and data protection

The study protocol was reviewed and approved by the institutional review board of the Comité d’é thique pour la recherche en imagerie médicale (Cérim; approval No. CRM-2006–099). Data processing steps were compliant with the European General Data Protection Regulation (GDPR).

## Declaration of competing interest

Kevin Maarek was an intern at Incepto Medical, the funder of the study.

Philippine Cordelle, Tom Vesoul, Pascal Zille, Gaspard d'Assignies, and Guillaume Herpe are employees of Incepto Medical, the funder of the study.

## Appendix Supplementary materials

Image, application 1
