# Supplementary material for: Enhancing knee MRI bone marrow lesion detection with artificial intelligence: An external validation study
Source: Res Diagn Interv Imaging. 2025 Aug 14;15:100063. doi: 10.1016/j.redii.2025.100063 (PMC12362699; doi:10.1016/j.redii.2025.100063)
Supplement: Supplementary file 1 [file mmc1.docx]

# **Supplementary Material**

Figure S1 illustrates bone marrow edema (BME) annotated by both experts using bounding boxes of two different colors (light blue: expert 1, green: expert 2). In this example, both experts annotated two areas of BME, identifying edema in anatomically similar regions of the knee. Although there are slight differences in the exact boundaries and extent of the annotations, both experts localized the edema within the same anatomical subregions. This consistency suggests a high level of agreement in the spatial identification of pathological regions, even when precise delineation may vary slightly between observers.


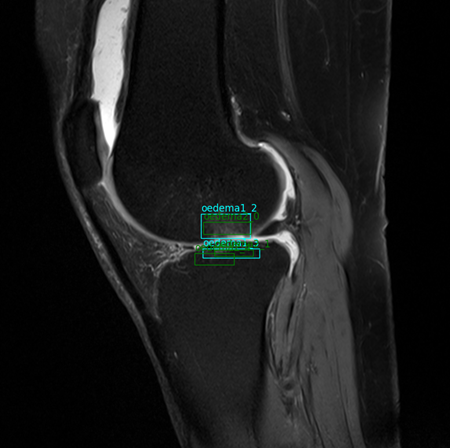


Figure S2 Illustration of the Genesis annotation platform displaying three DP FS sequences and the form to fill out with the detected bone edemas. The form on the right-hand side lists the 15 anatomical subregions of the knee, enabling the reader to simply check the boxes corresponding to the regions where bone marrow edema (BME) is observed. The platform provides the reader with MRI images in three orthogonal planes (coronal, sagittal, and axial), all displayed in fat-saturated sequences to enhance the visibility of BME. During the second phase of the reading exercise, the form is pre-filled with the AI-detected BME regions, and these regions are also outlined on the corresponding MRI images, allowing the reader to quickly review, confirm, or modify the AI’s annotations. In the first phase, readers did not have access to any pre-existing overlays (such as MOAKS) and were required to independently determine the locations of BME based solely on their visual interpretation of the MRI images. If a reader incorrectly localized the BME—by checking the wrong anatomical region—this was counted as a misclassification in the analysis of BME detection accuracy.


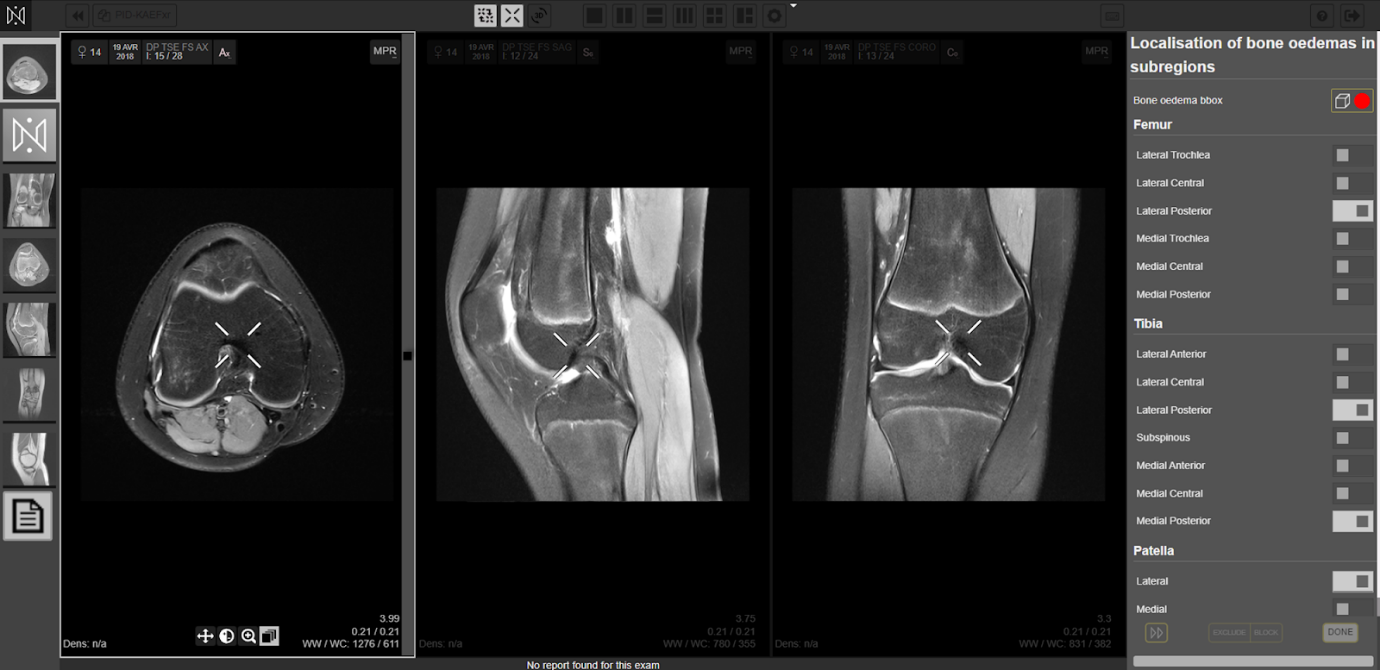


Table S1 illustrates the demographics of the training dataset

| **Demographics of the training Population of 1612 patients** | |
| --- | --- |
| **Female, n (%)** | 821 (50.9) |
| **Male, n (%)** | 791 (49.1) |
| **Age, mean (SD)** | 46.2 (18.4) |

Table S2 illustrates the magnetic field and manufacturers of the examination from the training and validation datasets

|  |  | **MRI Scans used for the training Dataset** | **MRI Scans used for the validation Dataset** |
| --- | --- | --- | --- |
| **Magnetic Field Strength, n (%)** | 1.0 | 194 (12.0) |  |
|  | 1.5 | 550 (34.1) | 65 (32.8) |
|  | 3.0 | 868 (53.8) | 133 (67.2) |
| **Manufacturers, n (%)** | GE MEDICAL SYSTEMS | 154 (9.6) | 15 (7.6) |
|  | PHILIPS | 1355 (84.1) | 102 (51.8) |
|  | SIEMENS | 103 (6.4) | 80 (40.6) |

Table S4 : Subgroup Statistical Analysis of BME detection of the 15 knee subregions without and with AI aid

|  | **Sensitivity without AI aid** | **Sensitivity with AI aid** | **Sensitivity p value** | **Specificity without AI aid** | **Specificity with AI aid** | **Specificity p value** |
| --- | --- | --- | --- | --- | --- | --- |
| **Femur LA** | 0.533 | 0.6 | 0.713 | 0.93 | 0.989 | 0 |
| **Femur LM** | 0.848 | 0.886 | 0.417 | 0.929 | 0.964 | 0.018 |
| **Femur LP** | 0.949 | 0.872 | 0.235 | 0.921 | 0.986 | 0 |
| **Femur MA** | 0.593 | 0.741 | 0.248 | 0.886 | 0.964 | 0 |
| **Femur MM** | 0.682 | 0.791 | 0.048 | 0.876 | 0.932 | 0.005 |
| **Femur MP** | 0.896 | 0.896 | 1 | 0.866 | 0.961 | 0 |
| **Patella L** | 0.912 | 0.93 | 0.728 | 0.882 | 0.938 | 0.002 |
| **Patella M** | 0.81 | 0.825 | 0.818 | 0.868 | 0.911 | 0.033 |
| **Tibia LA** | 1 | 1 | 1 | 0.874 | 0.94 | 0 |
| **Tibia LM** | 0.707 | 0.8 | 0.185 | 0.89 | 0.929 | 0.033 |
| **Tibia LP** | 0.919 | 0.904 | 0.669 | 0.871 | 0.919 | 0.024 |
| **Tibia MA** | 0.792 | 0.792 | 1 | 0.887 | 0.944 | 0.001 |
| **Tibia MM** | 0.883 | 0.933 | 0.343 | 0.893 | 0.935 | 0.017 |
| **Tibia MP** | 0.885 | 0.874 | 0.816 | 0.842 | 0.861 | 0.408 |
| **Tibia Subspinal** | 0.483 | 0.828 | 0 | 0.915 | 0.902 | 0.497 |
| **All** | 0.793 | 0.854 | 0 | 0.889 | 0.939 | 0 |

LA : lateral anterior LM : lateral medial LP : lateral posterior MA : medial anterior MM : medial medial MP : medial posterior

Table S5: Subgroup Statistical Analysis of BME detection performances of standalone AI subdivided by the 15 knee bone subregions

|  | **PPV** | **NPV** | **Specificity** | **Sensitivity** | **ROC AUC** |
| --- | --- | --- | --- | --- | --- |
| **Femur Lateral Anterior** | 0.75 | 0.988 | 0.994 | 0.6 | 0.793 |
| **Femur Lateral Middle** | 0.857 | 0.966 | 0.966 | 0.857 | 0.945 |
| **Femur Lateral Posterior** | 0.687 | 0.988 | 0.970 | 0.846 | 0.955 |
| **Femur Medial Anterior** | 0.727 | 0.994 | 0.982 | 0.888 | 0.933 |
| **Femur Medial Middle** | 0.745 | 0.941 | 0.914 | 0.813 | 0.8905 |
| **Femur Medial Posterior** | 0.867 | 0.982 | 0.988 | 0.812 | 0.966 |
| **Patella Lateral** | 0.518 | 0.968 | 0.921 | 0.736 | 0.920 |
| **Patella Medial** | 0.714 | 0.963 | 0.963 | 0.714 | 0.868 |
| **Tibia Lateral Anterior** | 0.6 | 1.0 | 0.988 | 1.0 | 0.988 |
| **Tibia Lateral Middle** | 0.567 | 0.972 | 0.899 | 0.84 | 0.918 |
| **Tibia Lateral Posterior** | 0.795 | 0.955 | 0.928 | 0.866 | 0.929 |
| **Tibi Medial Anterior** | 0.625 | 0.982 | 0.982 | 0.625 | 0.808 |
| **Tibia Medial Middle** | 0.791 | 0.993 | 0.969 | 0.95 | 0.968 |
| **Tibia Medial Posterior** | 0.658 | 0.986 | 0.909 | 0.931 | 0.942 |
| **Tibia Subspinal** | 0.571 | 0.964 | 0.883 | 0.827 | 0.886 |
| **All Anatomic Regions** | 0.698 | 0.977 | 0.952 | 0.834 | 0.923 |
